# Thermal Melanism in *Pachnoda iskuulka* (Coleoptera: Scarabaeidae: Cetoniinae)

**DOI:** 10.3390/insects16010061

**Published:** 2025-01-10

**Authors:** Petr Bogusch, Oto Petřík, Antonín Hlaváček, Ondřej Šebesta, Petr Šípek

**Affiliations:** 1 Department of Biology, Faculty of Science, University of Hradec Králové, Rokitanského 62, 500 03 Hradec Králové, Czech Republic; otopetrik@seznam.cz; 2 Department of Zoology, Faculty of Science, Charles University, Viničná 7, 128 44 Praha, Czech Republic; antonin.hlavacek69@gmail.com (A.H.); petr.sipek@natur.cuni.cz (P.Š.); 3 Department of Ecology, Faculty of Environmental Sciences, Czech University of Life Sciences Prague, Kamýcká 129, 165 00 Praha, Czech Republic; 4 Viničná Microscopy Core Facility, Faculty of Science, Charles University, Viničná 7, 128 44 Praha, Czech Republic; ondrej.sebesta@natur.cuni.cz

**Keywords:** coloration, beetle breeding, color ratio, polymorphism, habitat, mimicry

## Abstract

Thermal polymorphism, usually represented by thermal melanism (darker coloration in cooler habitats), is a well-known phenomenon in animals. In Cetoniinae, several species in captivity tend to become darker after several generations of breeding, which is probably caused by a lower temperature than is typical for their native habitats. *Pachnoda iskuulka* is a beetle species occurring in Somaliland. This species is easy to breed in captivity, and it is colorful and variable in the proportions of yellow, red, and black coloration. We kept this species from the first instar larva to the adult stage at three different temperatures. Elytra and pronotum of the adults were photographed, and proportions of the three main colors were measured. The proportion of black coloration significantly increased with size and decreased with temperature, while the proportion of yellow color increased. This species is certainly thermally polymorphic, which can be an adaptation for activation even at lower temperatures. The possible mimicry with beetles of the genus *Hycleus* is discussed. It is the first confirmation of thermal polymorphism in Cetoniinae and one of a few in Coleoptera.

## Simple Summary

Although thermal polymorphism is a quite widespread phenomenon in many groups of animals, there are not many examples known among beetles. In Cetoniinae, several species show color variability, which can be the result of different temperature conditions in their habitats. *Pachnoda iskuulka* is a colorful species that was described several years ago and currently has become one of the most favorite species in beetle hobby breeding. Incubation of larvae of this species at three different temperatures resulted in differences in the proportions of two main colors—yellow and black—on the body surface of adults. Proportions of both colors are significantly influenced by temperature, while the proportion of yellow is higher at higher temperatures. The situation with black is the opposite. Beetles reared at higher temperatures also have shorter development times. This example of color polymorphism is one of a few within the Scarabaeidae, and our methodology can lead other researchers working with related species to similar or different results.

## 1. Introduction

Polymorphism is a phenotypic variation along a continuum in which one species can exist in different forms [1]. This variability can have several causes: heredity, seasonality, sexuality (so-called sexual dimorphism or dichroism), crypsis, and many others. Polymorphism can also be caused by abiotic factors, e.g., by altitude and the associated variation in temperature at which a juvenile individual develops [2,3].

If the leading factor is temperature, we refer to thermal polymorphism. This is typically manifested by melanism, a significant increase in pigmentation of the body parts or suppression, respectively [4,5]. Thermal melanism is an adaptation that allows organisms to cope with colder environments, when the dark surface allows the body to heat faster under the influence of sunlight and to maintain a higher body temperature even at relatively lower ambient temperatures. However, this adaptation is usually only beneficial in habitats with sufficient solar radiation [5,6]. In addition, it is more critical for ectothermic animals, which obtain a major proportion of body heat from the surrounding environment and whose evolutionary success is mainly dependent on the use of available energy. The melanic coloring of the body can also mean the loss of the original cryptic or aposematic (warning) coloration and, thus, a higher risk of predation [5]. This trade-off is well known, e.g., in the European viper (*Vipera berus*), which loses both its cryptic and aposematic coloration through melanism [7].

In insects, thermal polymorphism is encountered in several cases. The migratory locust (*Locusta migratoria*) can take on different shades, depending on the temperature and color of the environment, ranging from a light color at high temperatures to almost completely black at very low temperatures [8]. In addition, in the common grasshopper (*Pseudochorthippus parallelus*), a reduction in some body protrusions (wings, limbs, etc.) was found in darker forms, which corresponds to Allen’s rule [9]. Another example is the common froghopper (*Philaenus spumarius*), for which a total of eleven different color forms with varying degrees of melanism have been described. An increase in the percentage of darker forms was noted at higher elevations [10]. Females are, on average, darker than males, possibly giving them an advantage in escaping predators; lighter, slower males are more likely to become preferred prey [11].

In Coleoptera, thermal polymorphism is well documented in ladybirds (Coccinellidae), especially in the two-spot ladybird, *Adalia bipunctata*, and the harlequin ladybird, *Harmonia axyridis* [12,13,14,15,16]. Thermal polymorphism, or melanism, in ladybirds is manifested by the extent of black spots; in extreme cases, by their absence or, conversely, complete coverage of the surface with a dark color [17]. *Harmonia axyridis* even exhibits thermal polymorphism of the pupa, which, in ladybirds, is exposed to natural influences. However, the intensity of coloration and color pattern of the pupa do not correlate with the coloration of the adult [18]. Polymorphism of coloration can also be found in *Chrysomela lapponica* (Chrysomelidae), which ranges from central to northern Europe. The study of Gross et al. [19] revealed that the extent of black-colored spots on the otherwise red elytra of beetles is greater in populations from Finnish Lapland than in those from climatically warmer Central Europe.

In the case of scarabs (Scarabaeidae), thermal polymorphism has only been studied in a few cases so far. Examples include the South African beetle, *Gymnopleurus humanus*, and the dung-eater, *Onthophagus proteus*, both species with iridescent coloration [20]. The polymorphism of these beetles is very likely influenced geographically, by latitude in *G. humanus* [21], or topographically, when the coloration of *O. proteus* is directly correlated with changes in altitude [22]. Polymorphic species could also be found in the subfamily Cetoniinae [23,24]. However, there are still no definitely confirmed cases of thermal polymorphism in this group.

The species we studied, *Pachnoda iskuulka*, comes from the Sanaag region in the northeast of Somaliland [25]. The sexual dimorphism of this beetle is unremarkable; males differ from females only by a shallow groove on the ventral side of the abdomen. The size varies from 24 to 28 mm. Based on studies of other species of the genus, there is a small but measurable difference in the size of males and females [26,27]. The beetles show color variability of the dorsal side, particularly on the pronotum and elytra (the head is always black). There is always a basal black spot on the back of the pronotum, which varies in shape and size and, in extreme cases, can extend anteriorly over the entire length of the pronotum to the head. The greatest variability can be observed in the elytra, the size and shape of the yellow spots on their basal parts, and the red-black edging of the middle and apical parts [25]. However, the function of its coloration is unknown and has not been studied.

In general, breeding animals in captivity allows us to observe how the biological characteristics of individual species change through time and from generation to generation [28,29,30]. This also specifically applies to insect breeding [27,31]. In the course of breeding, a tendency toward gradual melanization of coloration was observed in some species (*Pachnoda fasciata*, *P. interrupta*, and *P. leclercqi* (P. Šípek, unpublished observations).

In our study, we focused on polymorphism in *Pachnoda iskuulka*, using controlled breeding in captivity to assess the effect of different breeding temperatures during larval development on size, weight, sex ratio, time of development, and especially the color pattern of adults of this species.

## 2. Materials and Methods

### 2.1. Rearing and Breeding

**Figure 1:**
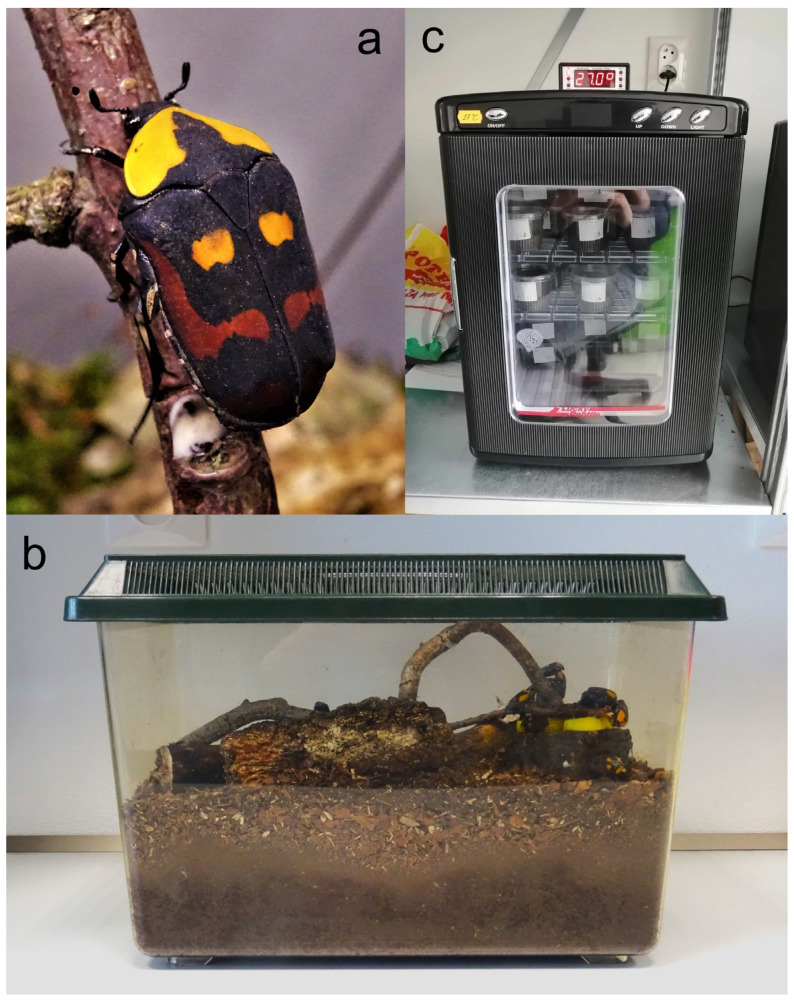
Female of *Pachnoda iskuulka* in cultivation terrarium (**a**), cultivation terrarium with adults (**b**), incubator with larvae in plastic boxes with leaf litter (**c**). Photos Oto Petřík.

In April 2020, ten fresh pairs of the second filial generation after wild-caught adults (F2) of *Pachnoda iskuulka* (Figure 1a) were provided for breeding and subsequent research. The beetles were inactive and, therefore, not yet mated. Maternal generation individuals were housed together in a 20-litre, 37 × 22 × 24.5 cm, Exo Terra faunarium with overhead ventilation (Figure 1b). In subsequent generations, beetles were reared separately according to the temperature at which they were incubated, i.e., adults from 24 °C in one vivarium, adults from 27 °C in another, etc. This method of division was applied throughout the remainder of the study. The boxes were filled approximately two-thirds full of crushed leaf litter. All boxes were illuminated with LED strips to simulate sunlight daily from 9 a.m. to 7 p.m. The three boxes were maintained under the same conditions.

The rearing conditions were set up according to the classical pattern of rearing beetles of the genus *Pachnoda*; the substrate humidity was medium to slightly drier (not measured) and replenished by a gentle sprinkling of water every day. In subsequent generations, following the note in [25] (higher activity even at temperatures around 40 °C), a heating element was added in the form of a heating stone, which locally increased the temperature at the point of contact up to 35 °C and significantly increased the egg-laying rate of females. The beetles were fed with ripe banana and salad cucumber every other day.

Breeding boxes were searched once a week, and hatched larvae were separated. The research took place in the form of controlled rearing of larvae in three incubators at different temperatures: 24 °C, 27 °C, and 30 °C. The temperatures should correspond with the temperatures of larval microhabitats in their native area of occurrence. Lucky Reptile Herp Nursery II incubators equipped with more precise thermostats (accuracy ± 0.1 °C) for more accurate temperature control were used. The humidity in the incubators was not regulated. The larvae were separated into perforated plastic trays with a volume of 250 mL. Crushed oak leaf litter was used as feed. The leaf litter came from the same source (near Chvaletice, Czech Republic, about 220 m above sea level) throughout the breeding period. The boxes were filled to approximately 90% of their volume. The breeding substrate was replaced when at least two-thirds of the substrate was visibly consumed, to provide a continuous source of nutrition (Figure 1c). Beetles were checked, and data were recorded (instars, pupation, deaths) weekly.

Larvae pupated in cocoons made of a mixture of leaf litter and excrements, which were usually glued to the wall of the box. To allow observation, the cocoons were placed on the surface of the substrate in a tub, and a hole with a diameter of approximately 2 mm was made in the wall with tweezers, through which the development of the larva inside was checked. This was done at least two weeks after finding the cocoon to avoid disturbance, which might cause abandonment of the cocoon.

Hatched adults were removed from cocoons and weighed on analytical balances after visible hardening and completion of coloration of the cocoons. This procedure was applied because it was observed that individuals that left the cocoon spontaneously after activation could already expel meconium. After measurement, the beetles were placed in breeding boxes and used for further breeding.

### 2.2. Photography and Image Analysis

Detailed pictures of the dorsal side of adult beetles were taken, using a tripod-mounted Canon 550D camera (Canon Inc., Tokyo, Japan) with a Canon MP-E 65mm f/2.8 1-5x Macro lens and illumination of the beetle from two sides by spotlights (OMLED-DP8W LED Dual Gooseneck Light). Data on the ratio of the three basic colors (black, red, and yellow) were digitally processed from these images. The ratio of their representation on the surface of the elytra and pronotum was a key variable for the observation of thermal polymorphism in *Pachnoda iskuulka*. Along with the photographs, a scale bar was included for measuring the dimensions of the beetle and the total area of the elytra and pronotum.

**Figure 2:**
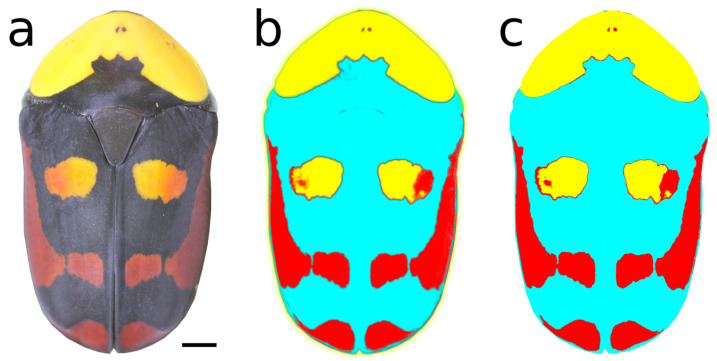
Example of original beetle image with manually cleaned background (**a**), the result of Ilastik machine learning segmentation: probability mask (**b**), areas made by thresholding of the probability mask image; the edges of the areas are the borders of the ROIs (Regions Of Interest) used for measuring (**c**). Scale bar represents 2 mm. Photos Ondřej Šebesta.

Photographs of beetles on a white background were hand-cropped to exclude protruding limbs, antennae, and problematic shadows (Figure 2a). All photos were taken with the beetle’s head oriented in the same direction. The pixel size of the individual photos was set according to the photographed scales (scripts in the attachments). Subsequently, randomly selected photos were used to train a machine learning algorithm for pixel classification so that the black and colored parts of the beetle and the white background were optimally classified, using the open-source software Ilastik (version 1.3.3post3). The output of this classification was a probabilistic map, according to which it was possible to segment colored spots, black parts and the whole beetle. The area of these regions was measured, and the percentage representation of yellow, red, and black was calculated (Figure 2b). The open-source software FIJI version 1.53t [32] with the “ilastik4ij” plugin (https://github.com/ilastik/ilastik4ij) (access date: 14 June 2023) was used to segment probability maps and measure areas (Figure 2c). 

While the black color is always sharply defined on the surface of the beetle, the red color transitions gradually into yellow almost imperceptibly. This factor must be considered due to the possible error rate of digital color measurement (red could be recorded as yellow at the transition point and vice versa). The variability of the shape and articulation of the spot on the pronotum were not assessed. Polymorphism in coloring was manifested by the extent of the black basal spot on the otherwise yellow pronotum and the ratio of the representation of black, red, and yellow colors on the rump. The ratio of the coverage of individual colors was measured from both parts as one whole (i.e., the pronotum and the elytra were not evaluated separately). The ventral side was not evaluated. Microscopy was performed in the Vinicna Microscopy Core Facility, Faculty of Science, Charles University, Prague.

### 2.3. Statistical Analysis

Statistical analysis and data visualization were performed in R software, version 4.3.1 [33]. The difference in the observed frequency of the sex ratios during different rearing temperatures and through generations was tested using goodness-of-fit tests based on χ^2^ statistics.

Development time under different temperature conditions was tested using the Kruskal–Wallis test by ranks, with post hoc Dunn’s non-parametric all-pairs comparison test for Kruskal-type ranked data, with a false discovery rate for *p*-value correction, using the PMCMRplus package [34]. We decided to use a non-parametrical comparison due to high differences in group variances, shown by Bartlett’s test.

In melanism analysis, first, we checked the distribution of variables and their collinearity, finding a high degree of collinearity between all colors (rpearson: −0.7 for black and yellow, −0.79 for black and red, and relatively lower for yellow and red, 0.13); thus, we focused only on one dependent variable: coverage of black color. We also found high collinearity between area of pronotum and elytra and body mass (rpearson = 0.58) and between the area and breeding time (rpearson = 0.21); hence, we excluded body mass and breeding time from further analyses and used only the area as a predictor variable; see Figure S1. We tested for associations between black color coverage (response variable) as a function of area, used as a continuous variable, generation, sex, and rearing temperature, used as categorical variables (predictors), using a linear model with Gaussian distribution. The final model predicting black color coverage was selected using stepwise selection using a partial F-test and acceptance threshold α = 0.05 at each step. Model assumptions were visually checked using the plot function. The effect of predictors on the black color coverage was tested using analysis of covariance (ANCOVA) followed by post hoc comparisons using the Tukey HSD method after checking homogeneity of variances using Bartlett’s test. We again checked the collinearity among predictors by estimating variation inflation factors (VIFs) using the vif function in the car v. 3.0-12 package [35]. In general, VIFs > 4 may indicate problematic collinearity [36]; we detected GVIFs < 1.06 in all cases. Variance explained by final model was in the model summary; variance explained by each of the predictors was counted as sum of squares of each predictor, divided by the total sum of squares.

Due to the extremity of the *p*-values, post hoc corrections for all performed models and test were not necessary.

## 3. Results

A total of 333 larvae were placed in controlled breeding in incubators, and 287 adults were successfully reared and measured, of which 147 were males and 140 were females. The material consisted of 102 beetles from the temperature of 24 °C, 94 beetles from the temperature of 27 °C, and 91 beetles from the temperature of 30 °C. The ratio of males and females slightly fluctuated within generation and incubating temperatures; however, frequencies did not significantly differ from the expected 1:1 ratio (rearing temperature: χ^22^ = 1.2593, *p* = 0.5328; generations: χ^23^ = 2.4988, *p* = 0.4755).

**Figure 3:**
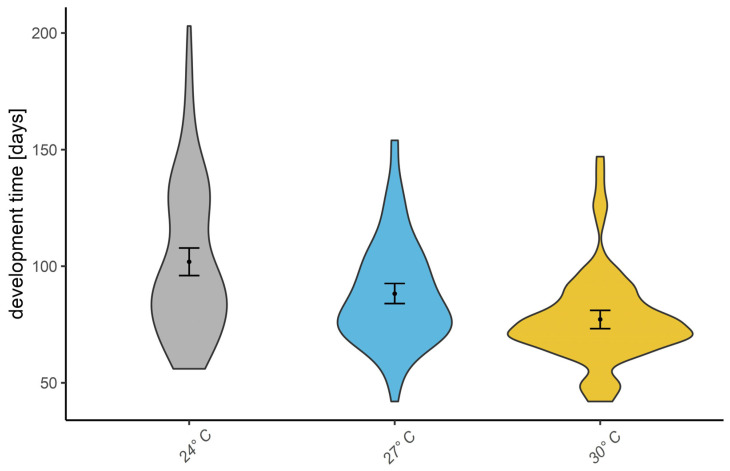
Development time of *Pachnoda iskuulka* adults reared from larvae incubated at 24 °C, 27 °C, and 30 °C. Post hoc Dunn’s non-parametrical test showed differences in all three groups (24 °C–27 °C: *p*-value = 0.0112, 24 °C–30 °C: *p*-value = 6.24 × 10^−10^, 27 °C–30 °C: *p*-value = 3.65 × 10^−4^). 95% bootstrapped confidence interval of mean and mean displayed.

While beetles reared at 24 °C took an average of 101 days to develop, those reared at 27 °C took only 88 days, and those reared at 30 °C took 77 days to develop. The Kruskal–Wallis test by ranks, with post hoc Dunn’s non-parametric all-pairs comparison test, showed significant differences between all groups (24 °C–27 °C: *p*-value = 0.0112, 24 °C–30 °C: *p*-value = 6.24 × 10^−10^, 27 °C–30 °C: *p*-value = 3.65 × 10^−4^); see Figure 3.

The measured surface of the elytra and pronotum of the beetles ranged from 131.53 to 248.45 mm^2^ (average 191.9 mm^2^). The highest proportion was covered by black color (mean 64.4% of the total measured surface), and it was the most stably represented color. The yellow color represented 19.39% and the red color 14.46% of the surface of the elytra and pronotum together.

We found that black coverage of the pronotum and elytra of *Pachnoda iskuulka* significantly positively correlated with the elytral and pronotum area (accounting for 27.7% of explained variability) and varied between breeding temperatures (accounting for 1.6% of explained variability); see Table 1. A small proportion of variability (3.2%) was absorbed by area when entered into the model, reflecting the overlapping contributions of the predictors. The proportion of black color differed only between beetles reared at 30 °C versus 27 °C (mean difference = −1.16, 95% CI [−2.24, −0.09], *p* = 0.030), with a lower proportion of black at the higher temperature. The differences for other factor combinations were not significant (24 °C versus 27 °C, mean difference = 0.57, 95% CI [−0.47, 1.62], *p* = 0.400), (24 °C versus 30 °C, mean difference = −0.59, 95% CI [−1.65, 0.47], *p* = 0.393). For mean values of color coverage during different breeding temperatures, see Table 2. No significant difference in black color coverage was found between males and females.

## 4. Discussion

Measurement of the proportions of colors on the surface of elytra and pronotum of *Pachnoda iskuulka* showed that with increasing temperature, the proportion of black color decreased significantly at the expense of the area occupied by yellow and red colors. No significant differences in coloration were demonstrated between males and females, although, on average, *P. iskuulka* males were darker than females. The black-colored parts made up the largest proportion of the measured surface of the dorsal side of the beetles, and the extent of the black color was the most stable, relative to the other colors, in the measured sample of beetles.

The increasing proportion of yellow color and the decreasing proportion of black color at higher temperatures supports the hypothesis of thermal polymorphism (melanism) in *P. iskuulka*. The proportions of black and yellow colors are most distinct at the highest temperature of 30 °C, while the colorations of beetles reared at 24 °C and 27 °C are more similar to each other. It is possible that at a lower temperature (for example, 21 °C or even 18 °C) the reared beetles would have a much larger proportion of black color and a smaller proportion of yellow color. Temperature itself accounted for 1.6% of the explained variability; however, an additional 3.2% of the variability was covered by the area of elytra and pronotum. The variance overlap suggests that both variables may capture similar underlying mechanisms. A dataset enriched with beetles reared at an even lower temperature, if tolerated by the larvae, could thus give clearer results [37]. Further research at higher temperature would probably incur a high larval mortality; even within the temperature range of the current study, larval mortality was higher at higher temperatures, although the difference was not statistically significant.

Based on data from the current study, the nature of thermal polymorphism in *P. iskuulka* seems to be similar to that of most invertebrates in which this phenomenon has already been studied. A similar effect has so far only been observed, but not quantified, in some related species (P. Šípek, unpublished observations). This polymorphism is, at first sight, difficult to discern, apparently due to its large variability even within individual temperatures. The thermal polymorphism of *P. iskuulka* shows results very similar to those from the much-studied beetle, *Chrysomela lapponica*. Adults of this beetle hatched at lower temperatures are darker and vice versa [19,38,39,40,41]. Melanism in *C. lapponica* is often attributed to geographic variability and the specialization of this species. In the case of *P. iskuulka*, these factors are likely less important because this beetle inhabits a very small area [25]. A similarity with *P. iskuulka* can also be found in the case of thermal melanism of different forms of ladybugs *A. bipunctata* and *H. axyridis* [12,13,14,15,16,42,43]. In *H. axyridis*, research on thermal melanism is complicated by the fact of non-random crossing of adults and the existence of several genetically stable color forms (f. *axyridis*, f. *conspicua*, f. *spectabilis*, f. *succinea*). No forms have yet been described for *P. iskuulka*, and due to the relatively low intraspecific color variability, the probability of this phenomenon in the case of this species is low.

Black and yellow showed the highest variability. Considering this, it is appropriate to consider the possibility of the occurrence of a more pronounced thermal polymorphism only in the area of the pronotum, which exhibits only these two colors. In this area, the extent of the black basal spots varies significantly. Variability in coloration limited to certain body segments can also be found in other groups of insects [44]. The black spot on the pronotum of *P. iskuulka* shows great variability in its shape and articulation [25]. However, since no suitable way of categorizing the black figures on the otherwise completely yellow pronotum was found, this method was not implemented in the current study. It is also possible that with a larger sample there would be almost smooth transitions between the theoretical “types” of spots. Further, comparison of pronotum and elytra separately could bring additional interesting results.

When considering color polymorphism, it should be noted that the entire captivity-bred population of *P. iskuulka*, bred both by scientists and by amateurs, is the offspring of only six females imported from Somaliland. The entire population thus underwent a significant bottleneck effect, which could have resulted in genetic drift and a change in the average coloration and its characteristics in the population [45]. The obtained results may not perfectly reflect the characteristics of the beetle in its native range. However, *P. iskuulka* is a species that naturally occurs in a relatively very small area, especially compared to other species of the genus [46] and is therefore less genotypically variable [47].

In its natural range, *P. iskuulka* was observed together with poisonous blister beetles of the genus *Hycleus* (Meloidae). The similarity of the color patterns of these two phylogenetically unrelated species of beetles is striking, and we assume this is a case of Batesian mimicry of *Hycleus* by *P. iskuulka*. The pattern of its coloring could show a correlation with the occurrence of certain species of extant poisonous beetles of the genus *Hycleus* (P. Šípek, unpublished observations). There is also the possibility of limiting color variability precisely by mimicry, since too much deviation from normal coloration for the purpose of better thermoregulation (e.g., a completely black beetle in a cold environment) would cause a potential loss of mimesis [48,49]. The polymorphism can be also connected with changes and differences of mechanical functions, such as flight balance, protection against predation, and structural support of studied body parts of adult beetles [50]. Environmental conditions, such as temperature, can certainly influence the material stiffness, flexibility, or overall durability of the elytra. Examining these effects may reveal broader evolutionary trends and adaptive strategies of studied species and enhance our understanding of the multifunctionality of elytra in relation to environmental adaptation.

Finally, it is necessary to mention the issue of the occurrence of melanism in insects. Since the synthesis and metabolism of melanins in insects is still poorly understood (in contrast to vertebrates), it is even more difficult to determine its formation as a manifestation of phenotypic plasticity in the case of thermal polymorphism [51]. According to [42], in the case of the eastern ladybug, the prepupal and pupal stages are critical for determining the final coloration. However, in the case of the ladybug, both the larva and the pupa are completely exposed to natural influences, while the larva and pupa of *P. iskuulka* are relatively hidden deep in the ground in a relatively stable environment.

## Figures and Tables

**Table 1:** ANOVA for LM on black color coverage of elytra and thorax, assuming a normal distribution of errors. The statistical significance of the variables was based on the F-ratio test. R^2^: 0.2941, Adjusted R^2^: 0.2867.

	DF	Sum of Square	Mean Square	F Value	*p* Value	R^2^
Area	1	1077.04	1077.04	111.36	2.0 × 10^−16^	27.7%
Temperature	2	63.59	31.79	3.29	0.03878	1.6%
Residuals	283	2737.10	9.67			

**Table 2:** Mean values (%) of color coverage at different breeding temperatures.

	24 °C	27 °C	30 °C
Black	64.98	64.94	63.22
Yellow	19	19.16	20.06
Red	14.38	14.11	14.92

## Data Availability

All data are available as electronic appendices.

## Supplementary Materials

The following supporting information can be downloaded at: https://www.mdpi.com/article/10.3390/insects16010061/s1, Figure S1: Correlogram of variables considered for use in the thermal melanism model of *Pachnoda iskuulka*, using Pearson correlation coefficient. Asterisks symbolize the correlation test results, and red lines loss functions; Table S1: List of all specimens with their weight and length of development; Table S2: List of all specimens with color proportions and ratios.
