# Supplementary material for: Thermal Melanism in Pachnoda iskuulka (Coleoptera: Scarabaeidae: Cetoniinae)
Source: Insects. 2025 Jan 10;16(1):61. doi: 10.3390/insects16010061 (PMC11765898; doi:10.3390/insects16010061)

Figure S1: Correlogram of variables considered for use in the thermal melanism model of *Pachnoda iskuulka*, using Pearson correlation coefficient. Asterisks symbolize the correlation test results and red lines loss functions.

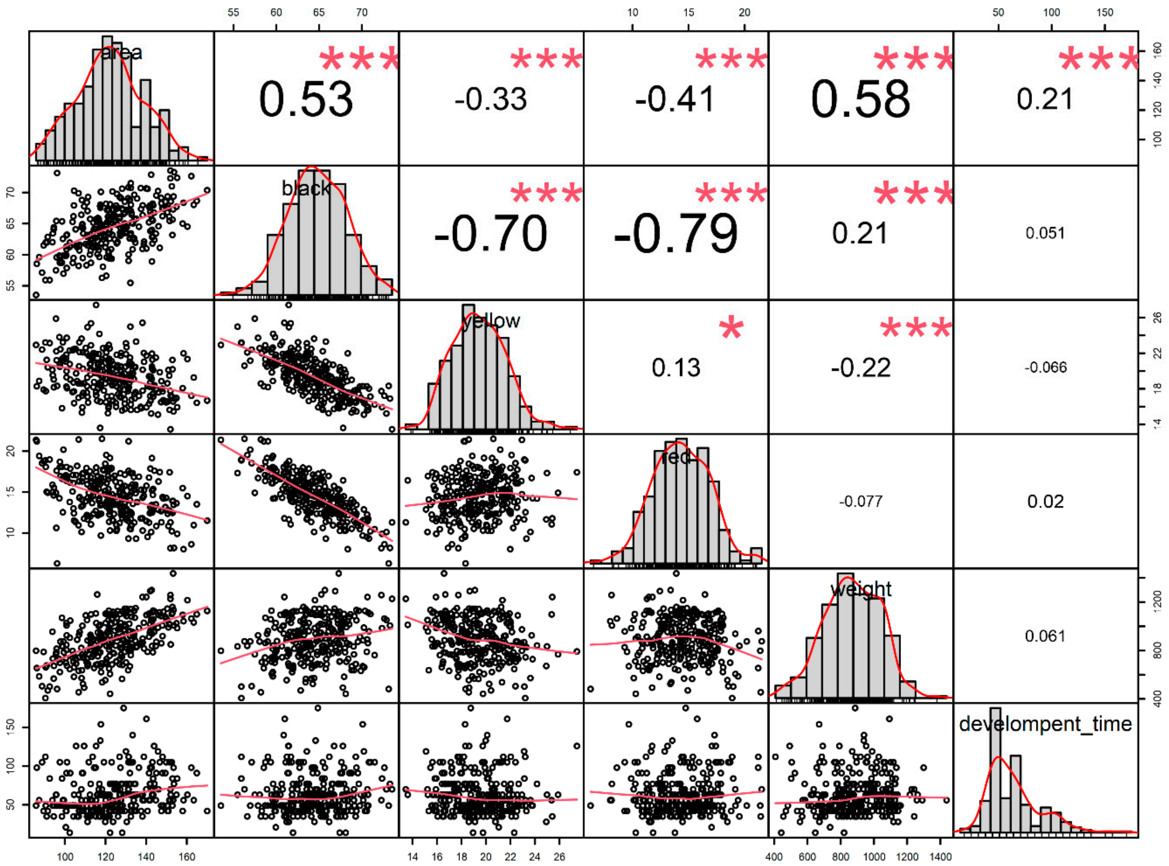

Supplement: Supplementary file 1 [file Figure S1_TRACKED.pdf › Figure S1_TRACKED.pdf]
